# Microfluidic study in a meter-long reactive path reveals how the medium’s structural heterogeneity shapes MICP-induced biocementation

**DOI:** 10.1038/s41598-022-24124-6

**Published:** 2022-11-15

**Authors:** Ariadni Elmaloglou, Dimitrios Terzis, Pietro De Anna, Lyesse Laloui

**Affiliations:** 1 Laboratory of Soil Mechanics, EPFL, 1015 Lausanne, Switzerland; 2 Laboratory of Environmental Fluid Mechanics, UNIL, 1015 Lausanne, Switzerland

**Keywords:** Civil engineering, Imaging techniques, Microscopy, Biomineralization

## Abstract

Microbially induced calcium carbonate (CaCO_3_) precipitation (MICP) is one of the major sustainable alternatives to the artificial cementation of granular media. MICP consists of injecting the soil with bacterial- and calcium-rich solutions sequentially to form calcite bonds among the soil particles that improve the strength and stiffness of soils. The performance of MICP is governed by the underlying microscale processes of bacterial growth, reactive transport of solutes, reaction rates, crystal nucleation and growth. However, the impact of pore-scale heterogeneity on these processes during MICP is not well understood. This paper sheds light on the effect of pore-scale heterogeneity on the spatiotemporal evolution of MICP, overall chemical reaction efficiency and permeability evolution by combining two meter-long microfluidic devices of identical dimensions and porosity with homogeneous and heterogeneous porous networks and real-time monitoring. The two chips received, in triplicate, MICP treatment with an imposed flow and the same initial conditions, while the inlet and outlet pressures were periodically monitored. This paper proposes a comprehensive workflow destined to detect bacteria and crystals from time-lapse microscopy data at multiple positions along a microfluidic replica of porous media treated with MICP. CaCO_3_ crystals were formed 1 h after the introduction of the cementation solution (CS), and crystal growth was completed 12 h later. The average crystal growth rate was overall higher in the heterogeneous porous medium, while it became slower after the first 3 h of cementation injection. It was found that the average chemical reaction efficiency presented a peak of 34% at the middle of the chip and remained above 20% before the last 90 mm of the reactive path for the heterogeneous porous network. The homogeneous porous medium presented an overall lower average reaction efficiency, which peaked at 27% 420 mm downstream of the inlet and remained lower than 12% for the rest of the microfluidic channel. These different trends of chemical efficiency in the two networks are due to a higher number of crystals of higher average diameter in the heterogeneous medium than in the homogeneous porous medium. In the interval between 480 and 900 mm, the number of crystals in the heterogeneous porous medium is more than double the number of crystals in the homogeneous porous medium. The average diameters of the crystals were 23–46 μm in the heterogeneous porous medium, compared to 17–40 μm in the homogeneous porous medium across the whole chip. The permeability of the heterogeneous porous medium was more affected than that of the homogeneous system, while the pressure sensors effectively captured a higher decrease in the permeability during the first two hours when crystals were formed and a less prominent decrease during the subsequent seeded growth of the existing crystals, as well as the nucleation and growth of new crystals.

## Introduction

During the past decade, microbially induced calcium carbonate (CaCO_3_) precipitation (MICP) has emerged as a sustainable alternative to traditional soil stabilization based on ordinary Portland cement^1^. MICP has been studied for a variety of potential engineering applications, such as soil improvement for increasing the stiffness and strength^2–4^ of granular soils, commonly referred to as biogrouting^5^; immobilization of heavy metals and radionuclides^6^; CO_2_ sequestration^7^ and sealing fractures in CO_2_ sequestration wellbores to mitigate leakage^8^. Ureolysis-based MICP, which is the most studied mechanism, occurs in two stages. In the first stage, ureolytic soil microorganisms, i.e., bacteria that secrete the enzyme urease, catalyze urea hydrolysis (Eq. 1). This reaction produces ammonium ions (NH_4_^+^) that increase the pH of the microenvironment, as well as carbonate ions (CO_3_^2–^). Consequently, alkalinity favors CaCO_3_ precipitation in the presence of sufficient calcium ions (Eq. 2):

1 $${\text{CO}}\left( {{\text{NH}}_{2} } \right)_{2} + {\text{H}}_{2} {\text{O}}\mathop{\longrightarrow}\limits^{{{\text{Urease}}\;{\text{enzyme}}}}2{\text{NH}}_{4}^{ + } + {\text{CO}}_{3}^{2 - } { }$$

2 $${\text{Ca}}^{2 + } + {\text{CO}}_{3}^{2 - } \to {\text{CaCO}}_{3} ({\text{s}}) \downarrow$$

The most commonly used microorganism in studies of MICP via ureolysis is *Sporosarcina pasteurii* (*S. pasteurii*) (strain designation ATCC 11859) because it is a nonpathogenic strain that has demonstrated a higher urease activity than many other strains^9^. *Sporosarcina pasteurii* belongs to the genus Bacillus, which is characterized as a Gram-positive bacterium that forms endospores and is aerobic^10^. The cells have a negative zeta potential that attracts calcium ions, thus favoring heterogeneous nucleation^11^. Heterogeneous or seeded nucleation refers to precipitation on surfaces such as cells, gas bubble^12^ and minerals^13,14^, which reduce the energy barriers that are required for nucleation and increase the nucleation rate. In contrast, homogeneous nucleation refers to CaCO_3_ precipitation from crystallization solutions. Therefore, the precipitation mechanism in MICP can be induced in two different ways: (i) by bacteria that act as nucleation sites, i.e., via epicellular precipitation, and (ii) by the increase in pH around the ureolytic cells due to the aforementioned urea hydrolysis reaction^15^.

The precipitated CaCO_3_ fills the pore spaces of granular media and, most importantly, endows them with true cohesion by bridging the soil grains, ultimately leading to increased shear strength and stiffness^2^. CaCO_3_ precipitation decreases the porosity and permeability of soils. The permeability decrease due to MICP treatment is less than 1 to 3 orders of magnitude in most cases, which is an advantage of MICP compared to chemical grouts that can reduce the permeability up to 8 orders of magnitude^16^.

The uniform delivery of reactants over distance is important for the remediation of large target zones in the field application of MICP^17,18^. A higher precipitation rate can lead to plugging near the injection point, while with a lower precipitation rate, higher uniformity can be achieved. Varying chemical concentrations of urea and calcium chloride in the Cementation Solution (CS) affect the precipitation rate and patterns causing microscale heterogeneities. The sizes, shapes and spatial distribution of the precipitated CaCO_3_ in the pore space can cause differences in the macroscale permeability and strength of the biocemented soils^19^. For example, Al Qabany and Soga^19^ showed that for highly concentrated CS of 1 M urea-calcium chloride^19^, larger crystals were formed that clogged the pores, leading to more rapid decrease of permeability and precipitation inhomogeneities. Another factor affecting the precipitation rate is the soil particle size and gradation^17^. CaCO_3_ precipitation is more effective at particle contacts^17^. Mortensen et al. ^17^ reported a higher rate of precipitation in dense well-graded and coarser sands than in loose, finer and poorly graded soils, which was attributed predominantly to the intrinsic properties of the base material subject to MICP.

The chemical reaction efficiency, which is defined as the percentage of injected calcium and urea that is converted to CaCO_3_, is an important parameter to control during the field-scale application of MICP to avoid the waste of raw materials^20^. Al Qabany et al.^21^ investigated the factors affecting the efficiency at the column scale, such as the chemical concentration in the CS, the treatment duration and the effective input rate, which was defined as the ratio of the chemical concentration to the no-flow time allowed for the reaction (the retention time). It was found that for an OD_600_ of 0.8–1.2, the effective input rate of 0.042 mol/L/h resulted in a reaction efficiency above 80% for all sand samples, independent of the chemical concentration used (0.25–0.5–1 M urea-calcium chloride). Zeng et al.^22^ reported a very low chemical efficiency of 5% in a field-scale experiment, which was attributed to preferential flow paths, channeling the reactants to specific zones of the soil matrix (sand lenses).

Traditionally, biocemented samples are observed through scanning electron microscopy (SEM), which requires destructive, posttreatment sampling to provide detailed qualitative information about the fabric and texture of the biocemented soils^23^. In recent years, experimental setups that combine microfluidics and imaging have been developed to probe microscale processes of MICP^23–29^ and Enzymatically Induced Calcite Precipitation (EICP)^30,31^ in real-time. Microfluidic devices integrate porous networks (known as a lab-on-a-chip) and facilitate imaging and tracking. They enable the visualization of transport processes at the microscale due to their transparency and small thickness and are commonly used for applications in biomedical research^32^, water monitoring research^33^, transport of bacteria in porous media^34^, and enhanced oil recovery^35^. Another technology that has been mobilized to study the nucleation and growth of single crystals is droplet microfluidics^36^. By using this setup, the authors were able to probe the encapsulation of cells by crystals, which is critical in terms of the availability of active cells for the continuation of ureolysis. Taking the above into consideration, the use of a lab-on-a-chip for MICP studies enables a series of real-time observations, including (i) quantification of bacteria, (ii) quantification of CaCO_3_ crystals, and (iii) determination of their shapes.

Xiao et al.^27^ used a Y-shaped microfluidic chip made of poly-dimethyl-siloxane (PDMS) with two inlet ports for the simultaneous injection of the bacterial suspension (BS) and CS that converged into a homogeneous pore-network reaction microfluidic channel. The images captured in an area of 2.8 mm by 4.5 mm (WxL) of the chip showed that CaCO_3_ precipitated first at the side of the injection of BS and then at the side of injection of CS. Moreover, the effect of different concentrations of calcium chloride on the diffusion and mobility of bacteria, the crystal growth kinetics and crystal distribution in MICP were explored in the study of Xiao et al.^29^. The results showed that a concentration of 0.5 M reduced the mobility of the bacteria, restricting them to the side of injection of BS. On the other hand, with a concentration of 0.01 M, a more homogeneous distribution of bacteria was achieved across the whole width of the chip at the end of the MICP treatment, which also led to a more homogeneous distribution of crystals.

Wang et al.^24–26,28^ used a PDMS microfluidic with dimensions of 15 mm by 15 mm, designed based on the cross-sectional images of a solidified and sectioned 3D Ottawa 30–50 sandy soil. Wang et al.^26^ demonstrated that a higher bacterial density resulted in an overall higher number of crystals and a faster crystal growth rate. More precisely, the highest bacterial density (~5.2 × 10^8^ cells/mL) yielded 2 × 10^7^ crystals/mL, with an average crystal volume of 450 μm^3^, while the lowest bacterial density (~0.6 × 10^8^ cells/mL) yielded a lower amount of crystals (1.1 × 10^6^ crystals/mL) but a higher average volume of 8000 μm^3^. Crystal growth was concluded at 1.5 h when the bacterial density was 5.2 × 10^8^ cells/mL, while a 10 times lower bacterial concentration resulted in 15 h of crystal growth. Wang et al.^28^ made the first published attempt to use the results from microfluidic experiments to optimize the column experiments. More specifically, the microfluidic experiments provided information about the size and number of crystals for low (3–5 h) and high (24 h) injection intervals that were also observed in the column experiments and were critical for the process efficiency of MICP and the mechanical strength of the treated soils.

Kim et al.^30^ used a homogeneous glass microfluidic device, with dimensions of 21.3 mm by 12.7 mm and explored the precipitation kinetics for multicycle treatment with EICP. A comprehensive image analysis algorithm to segment the pore structure and the precipitated CaCO_3_. To the best of our knowledge, the work by Kim et al.^30^ is the first one to compare the observed nucleation rate during the experiment in the micromodel, which simulates the not well-mixed conditions in a homogeneous porous medium, with the predicted nucleation rate based on the classical nucleation theory. Since microfluidics enables only 2D imaging, assumptions had to be made regarding the shape of the crystals (spherical and semispherical) to evaluate the crystal growth in three dimensions. Weinhardt et al.^31,37^ presented an experimental setup for the robust measurement of pressure in PDMS microfluidic channels of quasi 1D and quasi 2D pore structure geometries and few mm length during CaCO_3_ precipitation via EICP and observed the influence of the geometry on porosity–permeability relationship. Using X-ray microcomputed tomography (XRCT), which resolves the third dimension of the CaCO_3_ precipitates after the end of the EICP treatment, suggested that the spheroidal and frustum shapes can be used to calculate the volume of precipitated CaCO_3_ from 2D projections.

Although the above studies have visualized a variety of injection strategies of BS and CS with different injection intervals, bacterial densities and concentrations of CS in microfluidic domains of different geometries in real-time, they are limited to an area of a few mm^2^. However, MICP is a reactive transport phenomenon during which reactants are consumed at a distance from the injection point, and the rate of detachment of bacteria can differ with the distance. Moreover, pore-scale heterogeneity is expected to affect the spatial distribution of reactants due to zones of higher velocity that can transfer bacteria over a longer distance. To the best of our knowledge, the impact of pore-scale heterogeneity on the chemical reaction efficiency of MICP has not yet been studied.

The objective of this paper was to investigate the effect of pore-scale heterogeneity on the chemical reaction efficiency of MICP. Two types of meter-long microfluidics with the same initial porosity were fabricated: homogeneous and heterogeneous microfluidics. This work introduces a novel experimental setup for the evaluation of the chemical reaction efficiency and the comparison of the effect of the produced precipitated CaCO_3_ mass on the permeability of homogeneous and heterogeneous porous media. The precipitation of CaCO_3_ was monitored both temporarily and spatially at different locations along the meter-long path, offering insight into the pore scale. An image processing algorithm was developed to analyze the experimental images and detect the number of bacteria, as well as the nucleation and growth of individual and agglomerated precipitated minerals with respect to the reaction time and spatial distribution. Based on the processed images, a comprehensive statistical analysis was performed to estimate the distribution of the bacteria and crystals along the whole chip spanning more than ten thousand pores to identify distinctive precipitation patterns for the two geometries.

## Materials and methods

### Microfluidic chip

**Figure 1:**
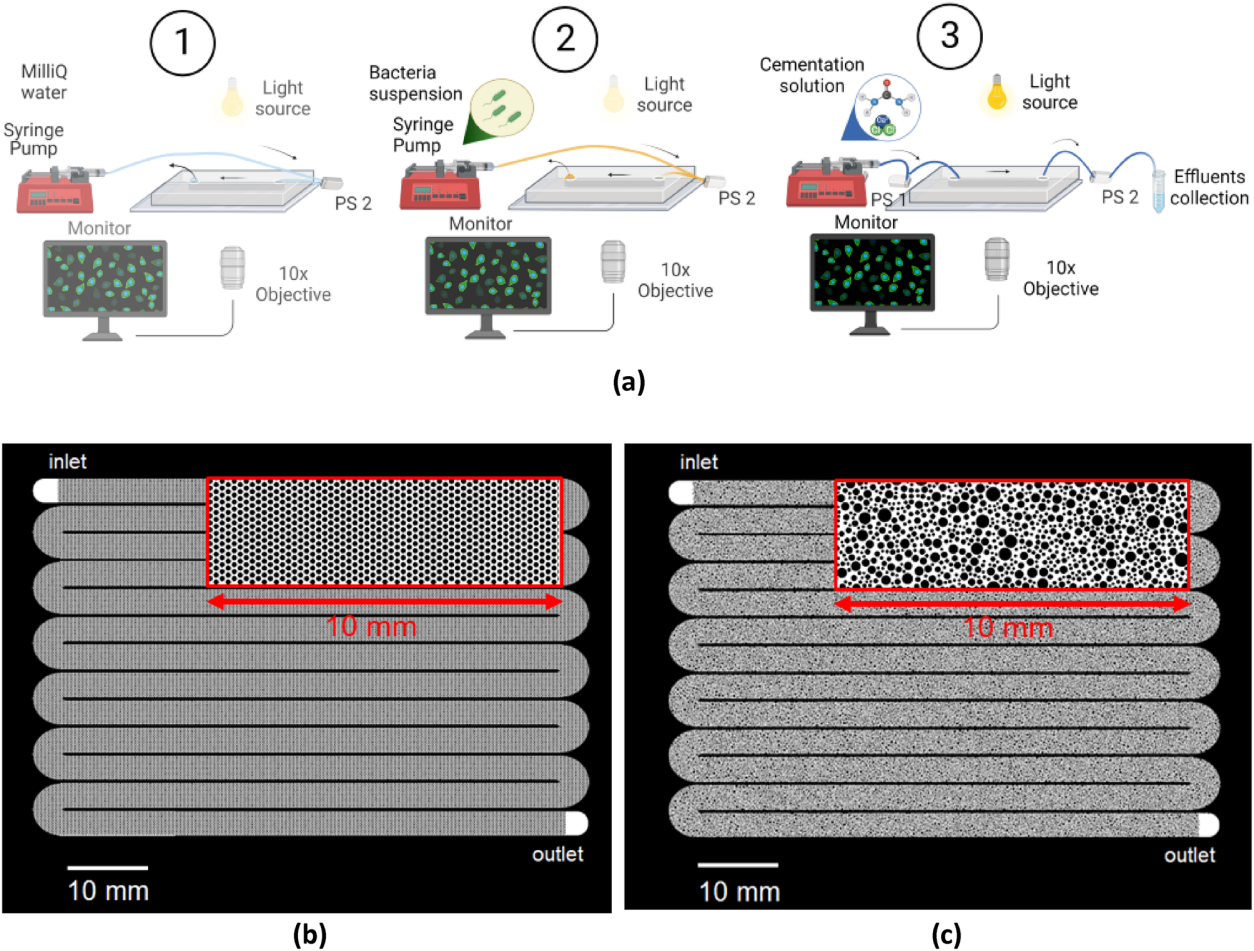
(**a**) Schematic representation of the experimental setup. The injection strategy involves (1) the injection of Milli-Q water from the outlet, (2) the injection of the BS from the outlet and (3) the injection of the CS from the inlet. PS1 and PS2 denote the pressure sensors connected at the inlet and outlet, respectively. Created with BioRender.com; (**b**) microfluidic design of a homogeneous porous medium; (**c**) microfluidic design of a heterogeneous porous medium.

To assess the effect of pore-scale heterogeneity, we developed a comprehensive experimental setup (Fig. 1a) that uses a very long microfluidic device that covers several orders of magnitude in average pore size that we fit onto a microscope glass slide of 75 mm by 50 mm, as shown in Fig. 1b, c. The same injection strategy was applied to two different microporous geometries with the same porosity (n = 0.5) in triplicate: one homogeneous (same pore size across the whole chip) and one heterogeneous, i.e., with spatially variable distances between grains. The first geometry consisted of a regular lattice of identical cylindrical solid grains, with a pore throat size (λ_1_) equal to 50 μm (Fig. 1b), while the second geometry consisted of irregularly distributed solid grains, with pore throat sizes ranging from 25 to 350 μm, with an average of λ_2_ = 70 μm (Fig. 1c)^38^. These artificial porous media are L = 990 mm long, W = 3 mm wide and H = 0.050 mm thick, corresponding to L = 19800λ_1_, W = 60λ_1_ and H = λ_1_ for the homogeneous porous medium and L = 14143λ_2_, W = 43λ_2_ and H = 0.7λ_2_ for the heterogeneous porous medium.

The microfluidic chips were fabricated using a standard soft photolithography process. The PDMS device was produced according to the protocol described by Karadimitriou and Hassanizadeh^39^. PDMS base was mixed thoroughly with curing agent in a 10:1 ratio. The mixture was subsequently degassed in a vacuum before casting into the mold and curing for at least 4 h in the oven at 60 °C. After removing the PDMS microfluidic channel from the mold, inlets and outlets of 1.2 mm diameter were punched. Both the PDMS chip and a glass cover were treated with plasma, which renders the PDMS surface hydrophilic^26^ like sand. Then, the chip was attached to the glass cover and placed on a hot plate at 100 °C for at least 30 min to ensure a strong permanent bond. Subsequently, the microfluidic chip was placed in a desiccator to degas the PDMS for approximately 30 min, which ensures the removal of any bubbles absorbed by the PDMS during chip saturation. As a gas slightly permeable material, PDMS allows for the aerobic growth of *S. pasteurii*. However, when experiments are run for a long time, the water can evaporate^40^. Therefore, an injection strategy was chosen so that the experiments ran for less than 24 h.

### Bacterial and cementation solutions

In the current study, liquid microbial growth medium (American Type Culture Collection (ATCC) 1376), which consisted of 20 g/L yeast extract, 10 g/L ammonium sulfate and 0.13 mol/L aqueous solution of Tris buffer with an initial pH of 9, sterilized separately and then mixed, was used. *Sporosarcina pasteurii* stored at − 80 °C was inoculated in 4 mL of fresh ATCC 1376 growth medium. The bacterial cells were grown in a shaking incubator at 180 rpm at 30 °C for 48 h to reach stationary conditions. The optical density (OD) was measured with a spectrophotometer at a wavelength of 600 nm (OD_600_). The bacterial suspension (BS) used in the microfluidic studies was obtained by diluting^41^ to reach an optical density of 0.4–0.55. A cementation solution (CS) was prepared by diluting equimolar concentrations (1 mol/L) of urea (CH_4_N_2_O) with a molarity of 60.06 g/mol and calcium chloride (CaCl_2_) (anhydrous Sigma–Aldrich) with a molarity of 110.98 g/mol in Milli-Q water.

### Injection strategy

The experiments were conducted in a well-controlled environment with a temperature of 25 ± 1 °C. Below, we explain in detail the injection strategy, which consists of three sequential injections: (i) saturation of the chip with MIlliQ water, (ii) injection of BS and (iii) injection of CS, during which the experimental setup is slightly modified as explained below (Fig. 1a). Injecting BS and CS using the same tubing would result in the mixing of the two solutions and subsequently in ureolysis and precipitation inside the tubing, which has a larger cross-section compared to the microfluidic setup. Thus, the resulting precipitation would not be representative of the spatial distribution across the 1 m distance. Therefore, the Milli-Q water used and the BS were injected from the outlet of the microfluidic channel (“outlet” in Fig. 1b, c), while the CS was injected from the inlet (“inlet” in Fig. 1b, c). High-precision pressure sensors (Elveflow MPS-S-1 with a working range of 340 mbar at the inlet and Elveflow MPS-S-0 with a working range of 70 mbar at the outlet) were placed in series to the flow near 7 cm from the inlet and 4 cm from the outlet. The sensors allowed the estimation of the pressure difference between the two points under flow conditions during CS injection, as well as during the no-flow retention and precipitation phase. The pressure sensors were fixed on the microscope stage with tape and connected to an Elveflow OB1 Pressure Controller^42^. The degassed microfluidic chip was placed horizontally on the stage.

Table 1 presents the composition of the BS and CS and the selected injection strategy. For the saturation of the microfluidic chip with Milli-Q water, the pressure sensor near the outlet was connected to the outlet and to a glass syringe (Hamilton) with Milli-Q water of 1–10 mL mounted to a syringe pump (Harvard Apparatus), while the inlet remained open to the air. For all connections, Tygon tubing 1/16″ was used. The chip was saturated with two pore volumes of Milli-Q water at a flow rate of 0.2 μL/s. At the end of the injection, a tiny water bubble was observed at the inlet so that air did not penetrate the microfluidic chip. Subsequently, a time interval of 30 min was allowed until the next step so that the gas bubbles were absorbed by the PDMS.

**Table 1:** The MICP injection strategy was applied in triplicate to the homogeneous and heterogeneous microfluidic chips.

Injected solutions	Flow direction	Number of pore volumes	Flow rate (μL/s)	Injection duration (min)	No-flow period (h)
Milli-Q water	Outlet	2	0.2	15	0.5
Bacterial suspension (OD_600_ = 0.4–0.55)	Outlet	6.7	0.2	40	1
Cementation solution (1 mol/L urea and calcium chloride)	Inlet	6	0.018	405	17

Following the full saturation of the microfluidic chip, the BS was injection from the outlet. For this purpose, the syringe containing the Milli-Q water was replaced by a glass Hamilton syringe of 5 mL containing BS. The bacterial injection of 500 μL (approximately 7 chip pore volumes of 75 μL) lasted for 40 min to achieve an approximately homogeneous distribution of the bacteria along the whole chip.

At the end of the bacterial injection phase, while the cells were settling, nineteen positions along the channel were selected for observations. This step lasted approximately 1 h, which was necessary to set the positions and focus of the 19 different locations. During this time, the cells grew from the initial OD_600_ of 0.40–0.55 before injection. Thus, we captured images at t = 0, which corresponds to the start of cementation injection and can represent the initial bacterial concentration. To inject CS from the inlet, first, the glass syringe connected to the outlet was carefully removed, and the outflow tubing was placed in a 50 mL Falcon tube for effluent collection. The Falcon tube contained 50 mL of Milli-Q water and was covered with parafilm and aluminum foil during both the injection and no-flow conditions to prevent evaporation and ensure a steady flow/no-flow, respectively. The urea hydrolysis started at the inlet as soon as the tubing containing the CS was connected to the chip device, already saturated with BS. Then, a glass Hamilton syringe of 10 mL was mounted on the syringe pump and connected through the pressure sensor to the inlet tubing (Fig. 1a). Before connecting the inlet tubing to the channel, it was fully saturated so that a liquid drop appeared at the pipe end to ensure full saturation upon connection. Cementation injection was performed with a flow rate of 0.018 μL/s, which corresponds to a seepage velocity of 0.24 mm/s, equal to the seepage velocity calculated for a laboratory-prepared sand sample with a height of 10 cm, diameter of 5 cm, and porosity of 0.4, which was injected at a flow rate of 10 mL/min^4^. The retention time of CS was 12–24 h. The applied flow rate corresponds to a Reynolds number of $Re = \frac{{\rho \overline{\lambda }\overline{u}}}{\mu } \ll 1$ (Table S1), where $\uprho$ is the water density (kg m^−3^), $\overline{\lambda }$ is the average pore throat size (m), $\overline{u}$ is the average fluid velocity (m s^−1^) and $\mu$ is the water dynamic viscosity (kg m^−1^ s^−1^). Therefore, the flow in the chip can be characterized as creeping flow, also known as Stokes flow, which is dominated by viscous forces. 6 chip pore volumes of CS were injected for 6.75 h, period during which crystal nucleation and growth was captured under the supply of fresh solution and continued during the retention period. For comparison, Xiao et al.^29^ reported a single injection step consisted of 7 chip pore volumes, while Kim et al.^30^ implemented a 10-cycle treatment in which each cycle consisted of about 100 pore volumes and was repeated at 48 h interval. Nevertheless, these works refer to much shorter chips and the injection of a single pore volume required only few minutes.

### Microscopy

**Figure 2:**
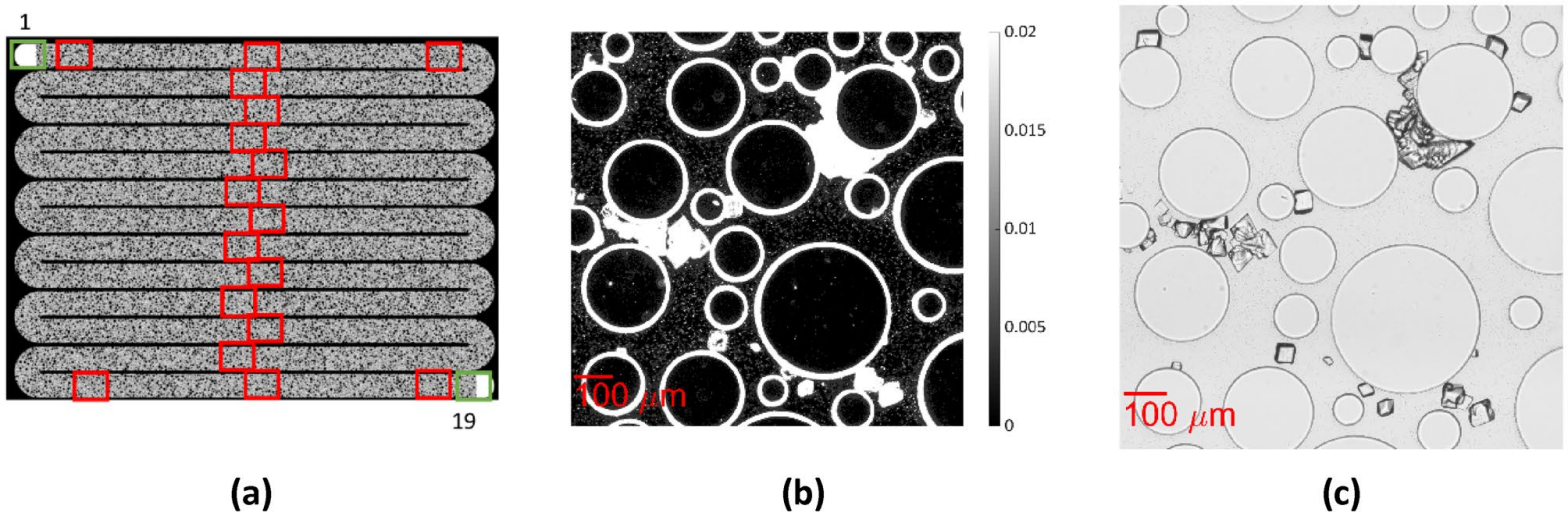
(**a**) Sampled positions; (**b**) Section of dimensions 1.022 mm × 1.022 mm of the green channel of the original image captured with phase-contrast microscopy and (**c**) of the green channel of the original image captured with phase-contrast microscopy.

Time-lapse imaging was conducted by a fully automated, inverted Nikon Eclipse Ti2 microscope (Plan Fluor 10 × Phase Contrast Objective) equipped with a Nikon DS-Ri2 Digital Colored Camera and controlled by NIS-Elements Software. Large images, composed of 9 tiled pictures with a size of 8813 pixels (2.57 mm in the transverse direction) by 13,252 pixels (3.87 mm in the longitudinal direction) and 3 × 3 fields were recorded at 19 positions along the path (Fig. 2a) with two different configurations, phase contrast (Fig. 2b) and brightfield (Fig. 2c). The 19 sampled positions included the inlet and outlet hole, 3 positions (left, middle, right) near the inlet and outlet, and in the middle of each row. The images were captured with modified phase-contrast microscopy (a ph3 ring combined with a ph1 objective so that illumination occurs almost laterally), resulting in a dark background with the bacteria being slightly brighter and the crystals appearing bright. In contrast, in the images captured with a brightfield microscopy configuration (without a condenser), the outline of the crystals appears dark, while the bacteria and the background appear bright. The camera exposure time was set to 50 ms for the phase contrast imaging and to 2 ms for the brightfield imaging. We used a high resolution of 0.29 μm/pixel to observe both the bacteria and crystals. As mentioned above, the positions were selected during bacterial settling, while the focus was made based on single immobile cells residing on the bottom of the chip (on the glass slide). The duration of injection of one pore volume of CS was approximately 62 min, while the acquisition system took 10 min to collect images from position 1 to position 19 with the two aforementioned optical configurations. To optimize the computational time of the large, collected data series, the time interval of image acquisition was 1 h during flow conditions and 2 h during the retention phase, since a slower growth rate was expected. Therefore, the temporal evolution of MICP in the microfluidic chip was monitored in real-time.

### Quantification algorithm

We developed a comprehensive image processing algorithm in MATLAB language to identify the precipitated CaCO_3_ and bacteria from the combined processing of the two types of raw images (phase contrast and brightfield).

A raw experimental image is a 24-bit RGB color image, consisting of three matrices 8813 × 13,252, each one representing the colors red, green and blue. Each matrix contains values ranging from 0 (black) to 255 (white). The three channels were extracted separately as 8-bit grayscale images with the use of NIS Elements software (Figs. S1, S2). We determined that the bacterial cells have the highest intensity in the green channel (Fig. S1), while the crystals also exhibit maximum absorbance in the green channel (Fig. S2). Thus, only the green channels of the acquired images were further processed to segment the solid medium structure (the grains), bacteria and precipitated CaCO_3_. After the connection of tubing at the inlet and the injection of CS, large bacterial aggregates were formed near the inlet due to positively charged calcium ions binding to the negatively charged *S. pasteurii* cells^26^ (Fig. S3). The intensity of these aggregates is similar to that of precipitated CaCO_3_; thus, the images captured closest to the inlet (x = ~5 mm) were not further processed.

As a first step, images from each position were selected at two different time points: (i) the beginning of the CS injection (t = 0), when only bacteria can be observed, and (ii) after the end of the CS injection (t = 10 h), when both bacteria and crystals could be observed. The images were normalized by 255 so that each pixel had values between 0 (dark pixel) and 1 (bright pixel). Below, we explain the workflow to detect the three phases: solid grains, bacteria and precipitated CaCO_3_ minerals.

#### Separation of solid grains

**Figure 3:**
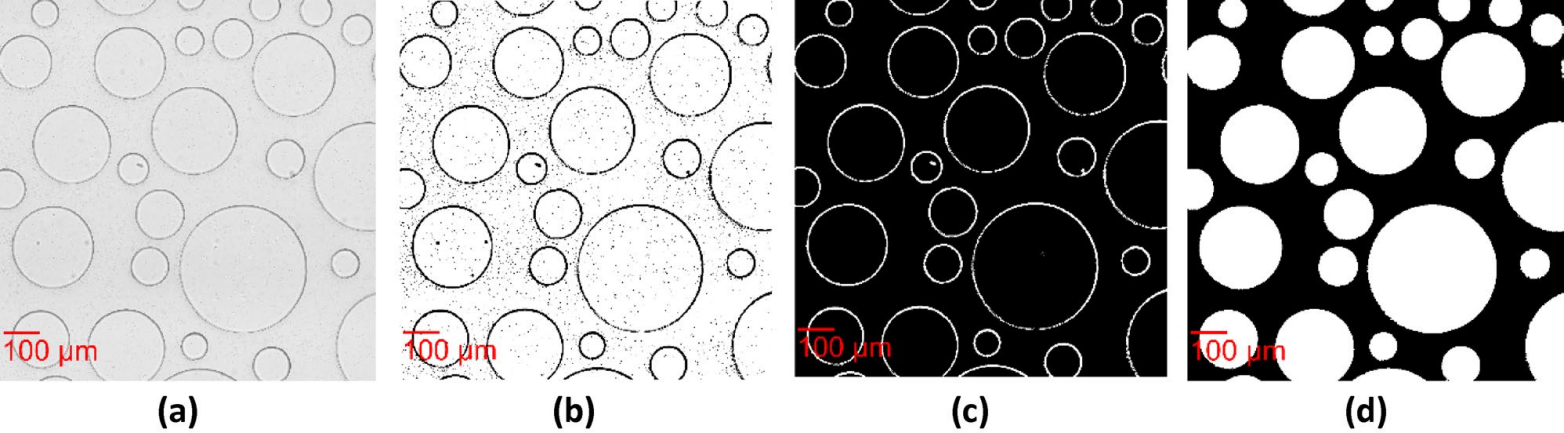
Workflow presenting the extraction of binary images of the pillars from the brightfield images at t = 0 (**a**) Section of dimensions 1.022 mm × 1.022 mm of the raw image of the green channel, (**b**) Image binarization, (**c**) Inversion and filtering of noise and bacteria with “bwpropfilt”, (**d**) Filling the pillars with white with “imfill”.

To separate the cylindrical solid grains (i.e., the circle in our images) from the brightfield image captured at t = 0 (Fig. 3a), the image was binarized with the "Imbinarize" function in MATLAB that replaces the values above an adaptive threshold with 1 (white) and the values below the threshold with 0 (black). This adaptive thresholding method selects a threshold based on the local mean intensity in the neighborhood of the pixel. The “ForegroundPolarity” “dark” option was used to detect the outlines of the pillars, as their intensity is lower than that of the background. The threshold was determined by the “Sensitivity” parameter, which can take values from 0 to 1 (see Supplementary material). By using a higher sensitivity, more pixels are defined as outlines of the pillars. The resulting binary image consists of the perimeter of the circular grains and bacteria, as well as noise inside the circular grains presented in black, and the pore space as well as the inside of the circular solid grains presented in white (Fig. 3b).

The pixels that represented bacteria and noise inside the circular grains were removed with the use of the "bwpropfilt" function on the inverse binary image. As a result, a binary image with the circles, corresponding to the perimeter of the pillars in white, and the pore space in black was created (Fig. 3c). Finally, the circular solid grains were filled with white color by using "imfill" (Fig. 3d).

#### Separation of the bacteria

**Figure 4:**
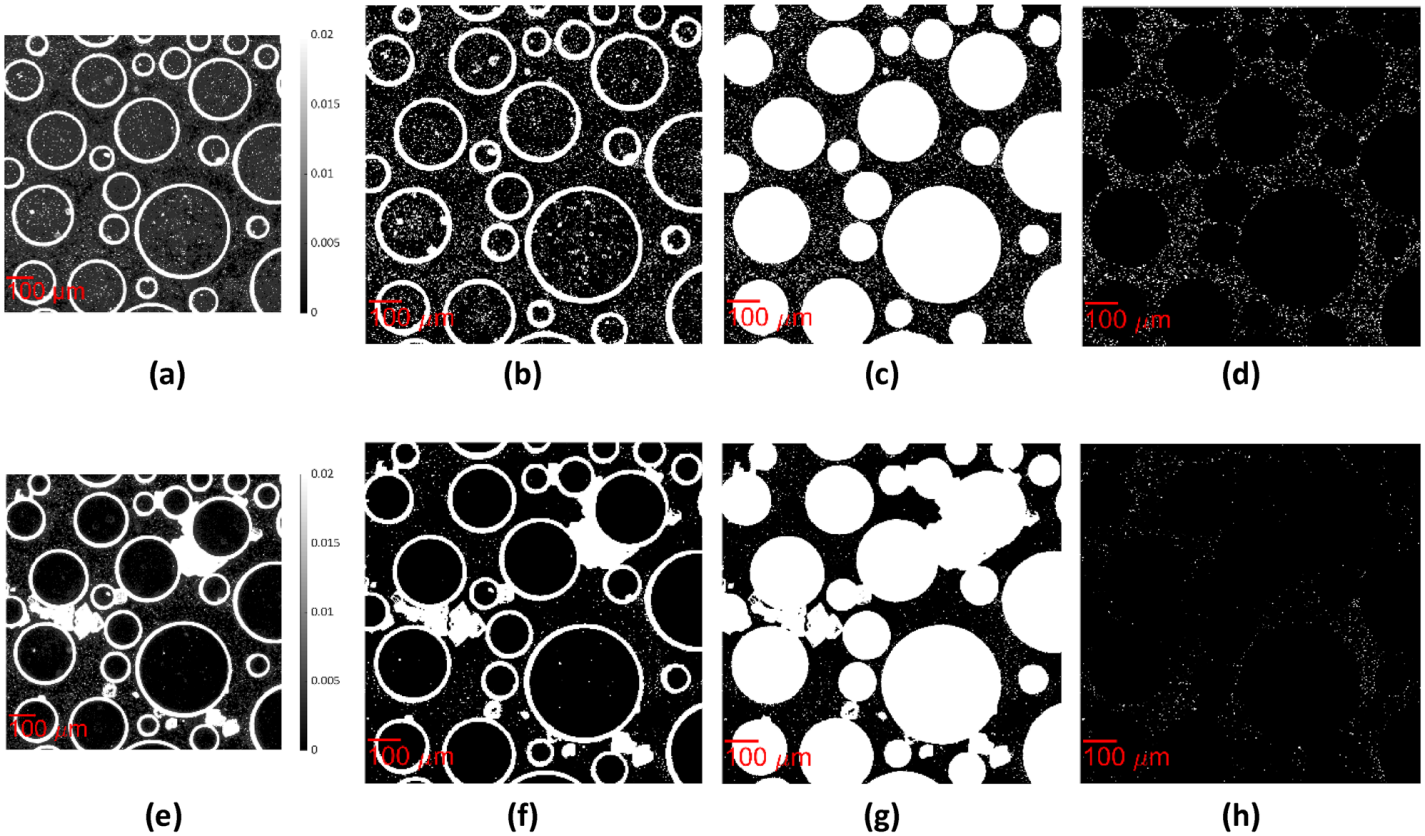
Workflow presenting the extraction of the binary image of bacteria from the phase contrast image at t = 0 (**a**) Section of dimensions 1.022 mm × 1.022 mm of the raw image of green channel; (**b**) Image binarization, (**c**) Set the pixels that correspond to the pillars in Fig. 3d to white, (**d**) Remove the pillars with “bwpropfilt”; at t = 10 (**e**) Raw image of green channel; (**f**) Image binarization, (**g**) Set the pixels that correspond to the pillars in Fig. 3d to white, (**h**) Remove the pillars with “bwpropfilt”.

The bacteria were separated from the phase contrast image captured at t = 0 (Fig. 4a). Each image was binarized with "Imbinarize" with the use of the adaptive thresholding method, setting the “ForegroundPolarity” parameter to the “dark” option. The value of “Sensitivity” was defined with trial and error between 0.1 and 0.2 to detect the bacteria, as their intensity is slightly higher than the background and much lower than the intensity of the perimeter of pillars and crystals. In the resulting binary image, the bacteria and the perimeter of the pillars are shown as white, while the pore spaces, as well as the inside of the circular grains, are shown as black (Fig. 4b). Subsequently, we set the pixels that correspond to the pillars from the previously acquired binary image in Fig. 3d to white (Fig. 4c). Finally, we separated the bacteria from the pillars by filtering the particles of area between 10 and 900 pixels^2^, corresponding to 2.9–261 μm^2^, as defined with Image Region Analyzer. A binary image where the bacteria appeared white on a black background was derived (Fig. 4d). The bacteria exhibited varying lengths because they grow after cell division (Fig. S4). The same procedure was followed to separate the bacteria at t = 10 h (Fig. 4f–h) from the phase contrast image captured at this timepoint (Fig. 4e).

#### Separation of the precipitated CaCO_3_

**Figure 5:**
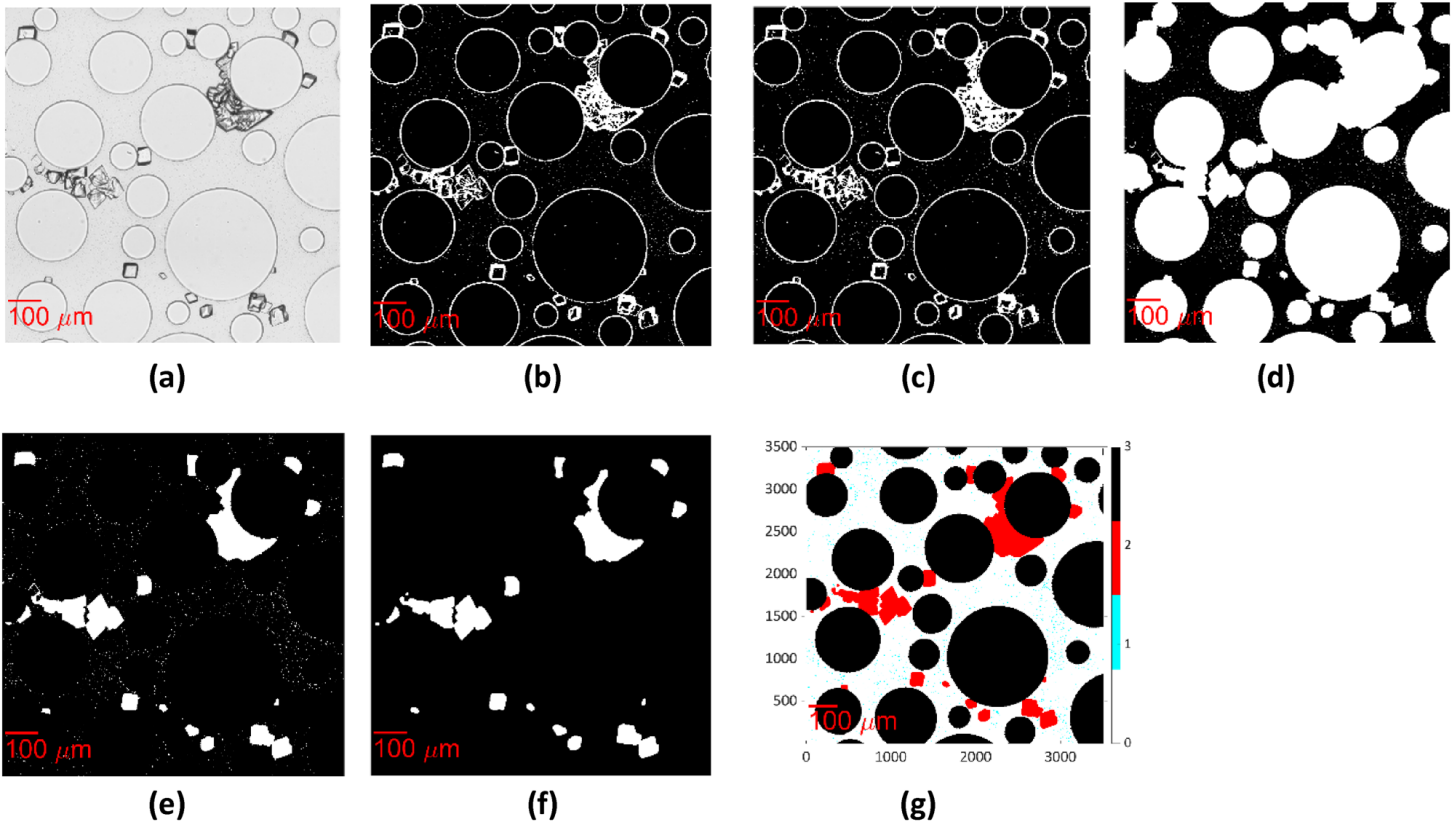
Workflow presenting the extraction of the binary image of crystals from the brightfield image at t = 10 h (**a**) Section of dimensions 1.022 mm × 1.022 mm of the raw image of the green channel; (**b**) Image binarization, (**c**) "Imclose" MATLAB function to smooth the edges of the outlines of crystals and close any existing holes, (**d**) “imfill” function to fill the crystals and the pillars with white (**e**) Set the pixels that correspond to the pillars in Fig. 3d to black, (**f**) Final result of the crystal segmentation (**g**) Final result of the image processing algorithm segmenting pore fluid (white), solid grains (black), and bacteria (cyan) at t = 10 h.

Subsequently, the precipitated CaCO_3_ was separated from the brightfield image captured at t = 10 h (Fig. 5a). The adaptive thresholding method was used, setting the “ForegroundPolarity” parameter in the “Imbinarize” to “dark” option. “Sensitivity” was defined with trial and error between 0.4 and 0.5 to detect the perimeter of the precipitated CaCO_3_. In the resulting binary image, the crystal perimeter, pillars and a few bacteria appeared white, while the pore fluid and the inside of the circular grains appeared black (Fig. 5b). The "Imclose" MATLAB function was used to smooth the edges of the outlines of crystals and close any existing holes (Fig. 5c). Then, we used the “imfill” function to fill the crystals and the pillars with white (Fig. 5d). Furthermore, the pixels corresponding to the pillars in Fig. 3d were set equal to zero. Then, the binary image of Fig. 3d was subtracted from this binary image to remove the pillars with their perimeter (Fig. 5e). Due to changes in illumination during the experiment, the outline of some pillars remained, and additional steps were required to remove them so that they would not be accounted for in the volume of the precipitated CaCO_3_ (see Supplementary Material). The separated CaCO_3_ phase is shown in Fig. 5f.

The final processed image produced by the algorithm presents the pore space in white (pixel value of 0), the bacteria in cyan (pixel value of 1), the CaCO_3_ in red (pixel value of 2) and the pillars in black (pixel value of 3) (Fig. 5g). With the use of this algorithm, the small crystals can be distinguished from the bacteria of the same size due to the smaller intensity of their perimeter in the green channel of the brightfield image.

#### Estimation of the bacterial concentration

The bacterial surface coverage was estimated by calculating the area of the pixels (with the MATLAB function “bwarea”) with a value of 1 in the binary image of the bacteria (Fig. 4d). Based on the reported size of *Sporosarcina pasteurii* cells of approximately 0.9–1.2 μm width by 2–3.5 μm length, and given that bacteria multiply by cell division^23^, for the following calculations we assumed that the diamensions of an individual bacterium are 4 μm length by 1 μm width.To calculate the number of bacteria, we divided the bacterial surface coverage by the average size of an individual bacterium of 4 μm^2^. The bacterial concentration was estimated by dividing the number of bacteria by the pore volume of each image, assuming a width of 1 μm in the 3rd dimension, due to the rod shape of *Sporosarcina pasteruii*^43^. The pore volume was estimated by subtracting the grain volume from the total volume of the sampled position. The grain volume in each sampled position was obtained by calculating the area of the pixels with a value of 1 in the binary image of the pillars (Fig. 3d) and multiplying by the depth of the microfluidic (50 μm).

#### Estimation of the chemical reaction efficiency of MICP

Assuming a spherical shape of the crystals^30,37^, their equivalent diameter D was defined as the average of their minor and major axes (single or aggregate); thus, their equivalent volume is $V_{cr,i} = \frac{4}{3}\pi \left( \frac{D}{2} \right)^{3}$. The total mass of precipitated CaCO_3_ at each sampled position was calculated:

3 $$m_{{{\text{CaCO}}_{3} }}^{{({\text{s}})}} = \rho \mathop \sum \limits_{\iota = 1}^{n} V_{cr,i}$$

where ρ = 2.71 g/cm^3^ is the density of calcite and n is the number of detected minerals (which can be both single crystals and crystal aggregates).

The chemical reaction efficiency E of MICP is defined as the ratio of the measured CaCO_3_ mass over the theoretical maximum value as if all the locally available calcium had reacted, assuming calcium is uniformly distributed along the channel. Hence the adopted setup allows to shed light onto specific precipitation patterns and answer questions such as: (i) is CaCO_3_ preferentially deposited in the vicinity of the inlet, where calcium is mostly available? (ii) does the pore network heterogeneity affects the deposition of CaCO_3_ across the reactive path.

The concentration of calcium in the injected CS is $C_{{{\text{Ca}}^{2 + } }} = c_{{{\text{Ca}}^{2 + } }} M_{{{\text{Ca}}^{2 + } }}$, where $c_{{{\text{Ca}}^{2 + } }} = 1\frac{{{\text{mol}}}}{L}$ is the molar concentration of calcium and $M_{{{\text{Ca}}^{2 + } }} = 40.08\frac{{\text{g}}}{{\text{L}}}$ is the molar mass of calcium. Thus, $C_{{{\text{Ca}}^{2 + } }} = 40.08\frac{{\text{g}}}{{\text{L}}}$.

According to the stoichiometry in chemical Eq. (2), 1 mol of ${\text{Ca}}^{2 + }$ reacts with 1 mol of ${\text{CO}}_{3}^{ - 2}$ to form 1 mol of CaCO_3._ Thus, 40.08 g/mol of injected ${\text{Ca}}^{2 + }$ can theoretically form 100.09 g/mol CaCO_3_ if all of it reacts with carbonate before being flushed out of the microfluidic chip.

The theoretical mass of the precipitated CaCO_3_ is:

4 $$m_{{{\text{CaCO}}_{3} ,{\text{t }}}}^{{({\text{s}})}} = { }M_{{{\text{CaCO}}_{3} }} \cdot Q \cdot \Delta t$$

where $M_{{{\text{CaCO}}_{3} }} = 100.09\;{\text{g/mol}}$ is the molar mass of CaCO_3_, $Q$ is the flow rate, and ${\Delta }t$ is the time interval since the beginning of the CS injection.

The local chemical efficiency at each position was calculated as follows:

5 $$E[{\text{\% }}] = \frac{{m_{{{\text{CaCO}}_{3} }}^{{({\text{s}})}} .PV_{l} {/}PV_{TOT} }}{{m_{{{\text{CaCO}}_{3} ,{\text{ t }}}}^{{({\text{s}})}} }}100$$

where $m_{{{\text{CaCO}}_{3} ,{\text{t }}}}^{{({\text{s}})}}$ is the estimated total mass of the precipitated CaCO_3_ at each sampled position calculated by (3), $PV_{l}$ is the pore volume at the specific position, and $PV_{TOT} = 74\;\upmu {\text{L}}$ is the total pore volume of the microfluidic channel. Each position corresponds to approximately 0.3% of $PV_{TOT}$. At the end of the CS injection, the theoretical mass of the precipitated CaCO_3_ is $44.3\;{\text{mg}}$ in the whole chip, while at each sampled position it is 110–130 μg depending on the locally available pore space.

## Results and discussion

### Spatial distribution of bacteria

**Figure 6:**
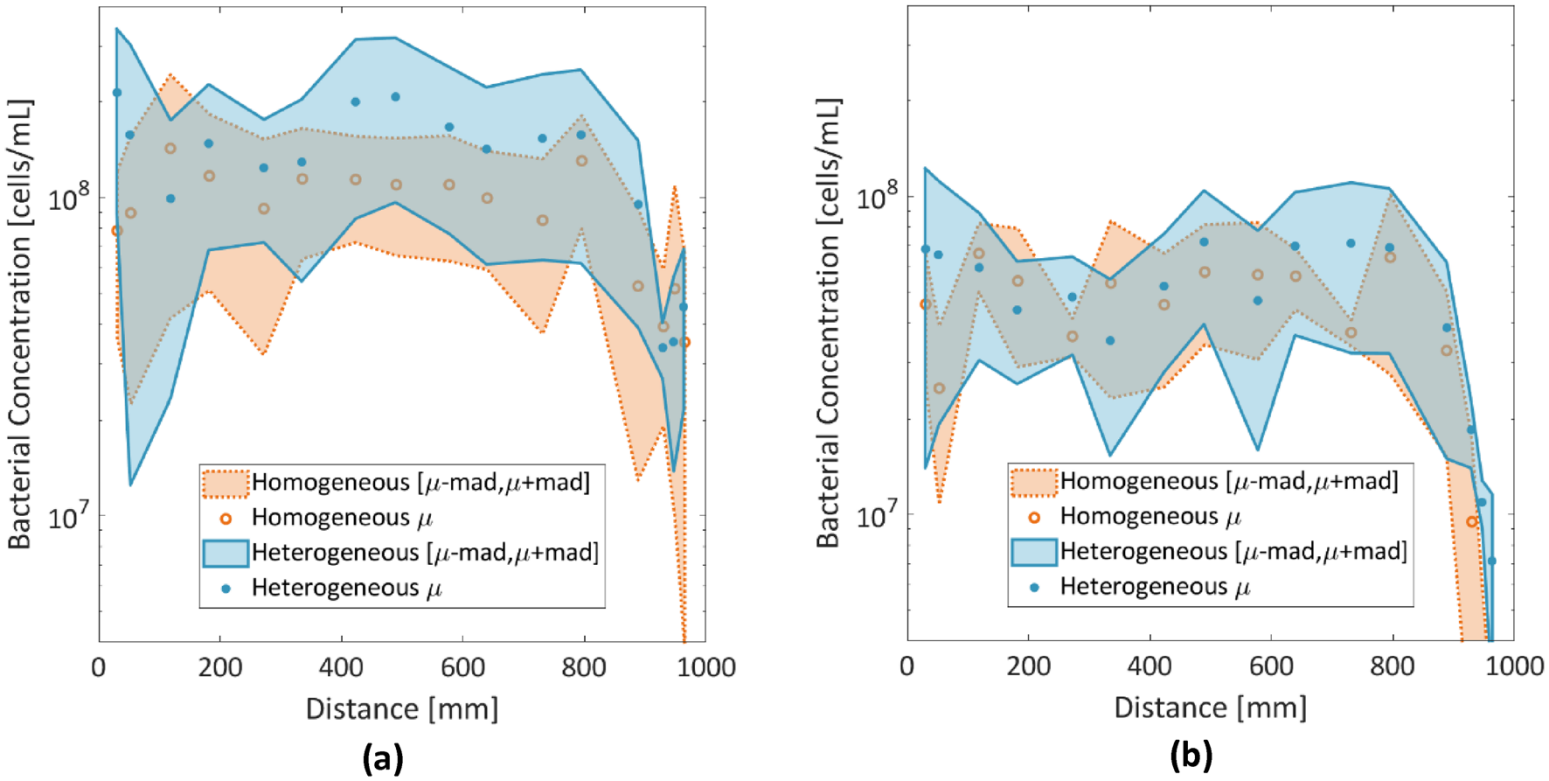
(**a**) Bacterial concentration of *S. pasteurii* at the beginning of CS injection (t = 0); (**b**) at t = 12 h across the meter-long reactive path. Dots represent averages of the triplicates; shaded areas represent the mean absolute deviation of the triplicates.

The bacterial density is believed to affect the growth rate, size and number of CaCO_3_ precipitates^26^. Figure 6a presents the bacterial concentration along the chip averaged over the triplicates at the beginning of the CS injection (t = 0) (Fig. 6a) and at t = 12 h (Fig. 6b). At t = 0, the average concentration of bacteria is more uniform in the homogeneous medium than in the heterogeneous porous medium at 0–800 mm, ranging from ~8 × 10^7^ cells/mL to 1.03 × 10^8^ cells/mL In the heterogeneous porous medium, the bacterial concentration decreases from ~2 × 10^8^ cells/mL to 1 × 10^8^ cells/mL in the first 400 mm and it presents a peak at 420 mm before it drops to ~1–1.05 × 10^8^ cells/mL in the interval 600–800 mm, where it remains approximately 50% higher than the concentration in the homogeneous porous medium. Close to the outlet, a lower bacterial concentration of ~3 × 10^7^ cells/mL to 4 × 10^7^ cells/mL was detected in both the homogeneous and heterogeneous microfluidic systems.

**Figure 7:**
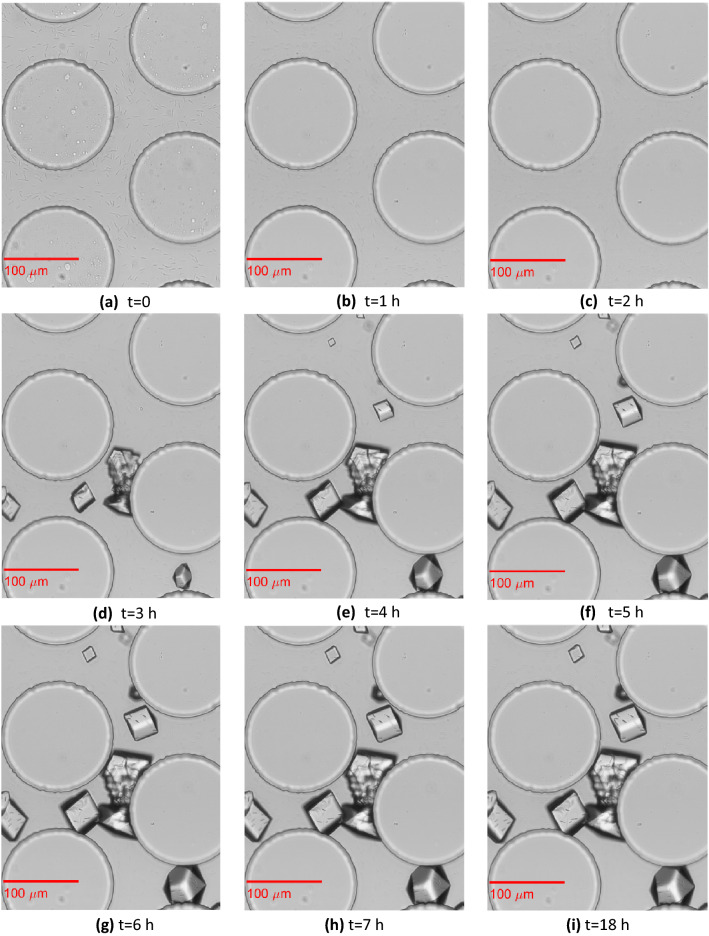
The growth of single crystals and crystal aggregates in selected pores of the homogeneous microfluidic chip captured ~423 mm from the inlet.

During CS injection, bacteria that were not attached to the pillars or the glass slide were transported or encapsulated in CaCO_3_ crystals (Fig. 7). At t = 12 h, a slightly higher average bacterial concentration of ~3 × 10^7^ cells/mL to 7 × 10^7^ cells/mL was retained by the heterogeneous porous medium than by the homogeneous medium, where an average concentration of ~2.5 × 10^7^ cells/mL to 5 × 10^7^ cells/mL was observed in 0–900 mm, while close to the outlet, most of the bacteria were flushed out.

### Crystal nucleation and growth

**Figure 8:**
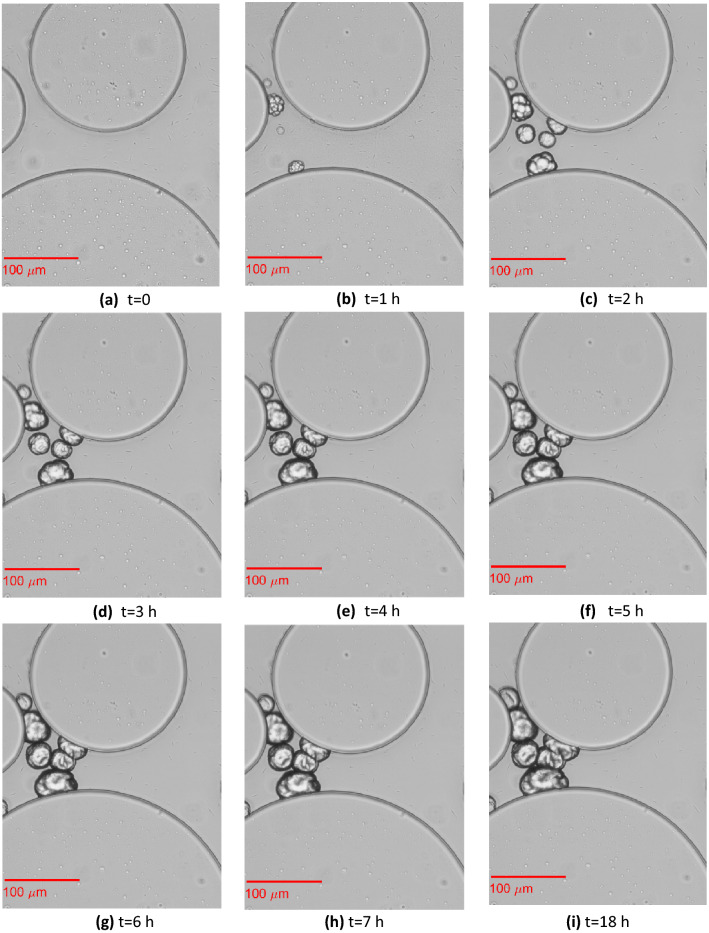
Evolution of crystal aggregation in the pore space among three cylindrical solid grains of the heterogeneous replica of a porous medium. This pore is located in a sampled location ~730 mm from the inlet.

Images of crystal growth in the selected pores of the homogeneous and heterogeneous microfluidics chips are shown in Fig. 7 and Fig. 8, respectively. Crystal nucleation started 1 h after the beginning of CS injection in both the homogeneous and heterogeneous porous media. Figure 7 shows single CaCO_3_ crystals formed 3 h after the beginning of CS injection ~423 mm from the inlet. Crystals nucleated both on the pillars and on the glass slide, where the bacteria remained attached. Initially, all crystals exhibited a size that was not large enough to bridge the adjacent solid grains. At t = 4 h, five more single crystals (two are out of focus) were formed, while the existing crystals grew larger. One of the single crystals grew large enough to bridge the two adjacent particles. Two other crystals merged and formed a crystal aggregate that also bridged two adjacent solid grains. At t = 5 h, all crystals grew larger. However, no further crystal growth was observed in this selected position during the following 1 h and 40 min periods of continuous CS injection or during the no-flow period until t = 18 h.

**Figure 9:**
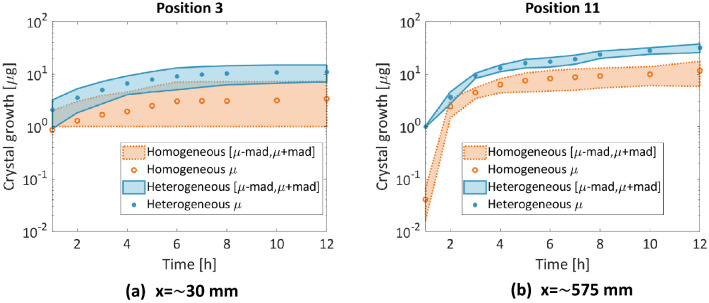
Crystal mass growth during 12 h of MICP treatment at two positions: (**a**) x = 30 mm and (**b**) x = 574 mm along the homogeneous and heterogeneous porous medium. Dots represent averages of the triplicates; shaded areas represent the mean absolute deviation.

Figure 8 shows four small single CaCO_3_ crystals that formed 1 h after the beginning of CS injection. As in Wang et al.^13^, single nucleation points that are close to each other grew separately until they merged to form crystal aggregates (mesocrystals^2^). Here, most of the growth occurred within the first 4 h, while it continued with a very slow rate until 18 h. This trend can also be verified by the plots of the mass crystal growth (Fig. 9). This figure presents the growth of the crystal mass at two positions: (a) close to the inlet and (b) in the middle of the microfluidic. It can be observed that the growth was overall higher in the heterogeneous porous medium. In both porous media, most of the growth was observed within the first 4 h. However, in the heterogeneous porous medium, the growth rate in the following 9 h of MICP treatment was higher than that of the homogeneous porous medium that presented a plateau. Crystal growth was completed 5 h (t = 12 h) after the end of the 6 h 40 min CS injection in most cases.

The distribution of precipitated CaCO_3_ in the transverse direction of flow varies from position to position, with crystals occupying the whole plane or close to the boundaries (Fig. S5). This can be attributed to CaCO_3_ precipitation altering the flow path or preferential paths that bring the reactants into zones where the mixing is enhanced. However, experiments with dyes could provide more insigths to the flow regime in the two chip and how it affect the mixing of reactants in the evolving pore space.

### Evaluation of the reaction efficiency

**Figure 10:**
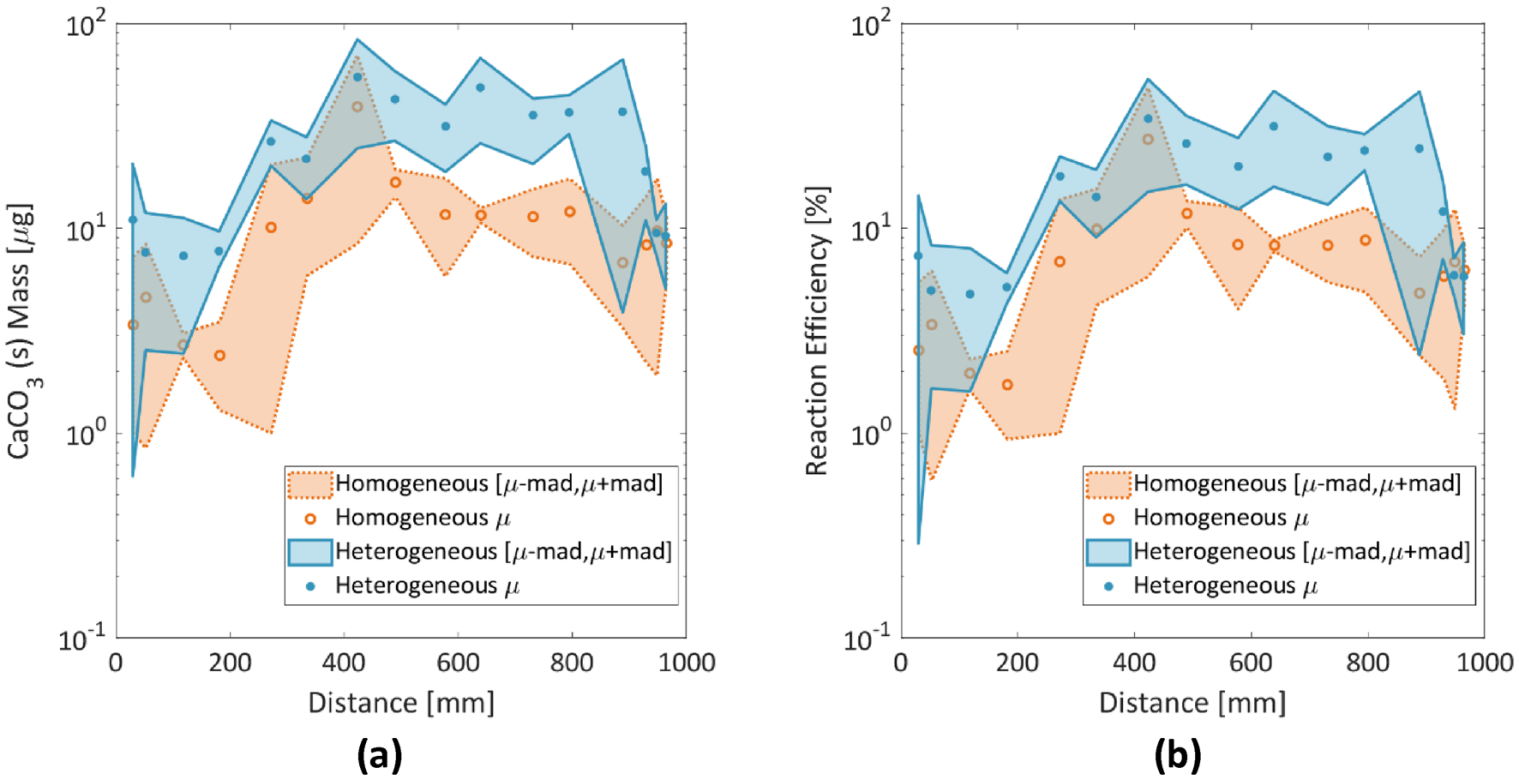
(**a**) Final mass of the precipitated CaCO_3_; (**b**) estimated reaction efficiency at the end of the MICP treatment (t = 12 h) when crystal growth was completed. Dots represent the averages of the triplicates; shaded areas represent the mean absolute deviation of the triplicates.

The chemical reaction efficiency in the present work is defined as the percentage of injected calcium that converts to CaCO_3_. The estimated mass of the precipitated CaCO_3_ presents a peak in the middle of the microfluidic channel for both homogeneous and heterogeneous porous media (Fig. 10a). Figure 10b presents the estimated chemical reaction efficiency in the homogeneous and heterogeneous porous media. Both the homogeneous and heterogeneous porous media present a nonuniform reaction efficiency with respect to distance. In the homogeneous porous medium, the efficiency is low near the inlet and it reaches a maximum at 420 mm with an average of 22%, while it remains below 11% between 600 and 990 mm. In contrast, the heterogeneous porous medium presents a peak value of reaction efficiency of 37% with a higher standard deviation at the middle of the microfluidic, and it yields values between 15 and 28% at distances between 500 and 900 mm from the inlet. This finding is further accompanied by a smaller deviation for the heterogeneous porous network after the triplicates were analyzed. We postulate that the discrepancy between the measured efficiency values in the present study and those reported in literature for lab scale samples, typically above 40%^2^ is due to the higher availability of surfaces in 3D column experiments where the grain characteristics, such as roughness^44^ or particle angularity and more complex threedimensional flow paths favor cell attachment and calcite precipitation.

**Figure 11:**
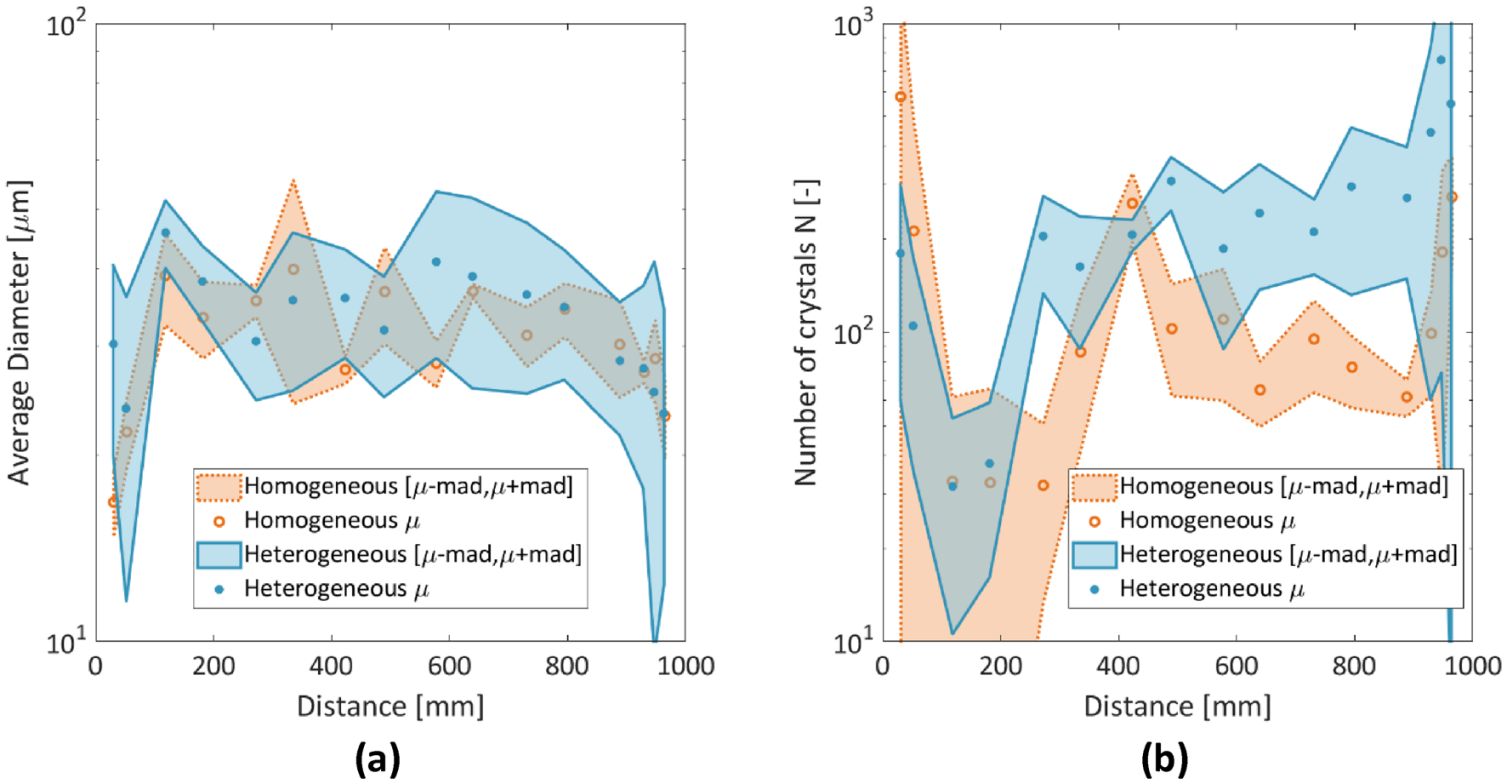
(**a**) Average diameter of crystals. (**b**) Final number of crystals at t = 12 h. Dots represent the averages of the triplicates; shaded areas represent the mean absolute deviation of the triplicates.

Based on the above findings, a preliminary observation is that by using opposite ends for the introduction of BS and CS, biocementation increases in the middle of the chip and subsequently evolves toward the CS flow path. However, the architecture of the flow network itself appears to influence this evolution in terms of reaction efficiency and crystalline properties. One possible explanation is that the predominant CaCO_3_ trend in the homogeneous porous network is that of small single crystals that do not grow enough to clog the pore throats and eventually attract further reactants, which results in lower efficiency. For the heterogeneous porous architecture, the trend is different, with various nucleation points that grow into larger aggregates (Fig. 11), and the clogged pore throats therefore grow in size and influence dynamically the network’s tortuosity.

These distinctive trends are further illustrated in Fig. 11a, b, where crystal numbers and sizes vary for the two chips. The crystals appear to be larger in the heterogeneous medium ($D_{eq}$ = 35–40 μm) than in the homogeneous medium ($D_{eq}$ = 28–35 μm) between 600 and 800 mm and yielded a larger mean absolute deviation at the second half of the chip (Fig. 11a). Furthermore, a larger number of crystals (200–300 crystals) were captured in the heterogeneous porous network between 480 and 900 mm, which reflects a higher calcite mass and efficiency compared to the homogeneous network (with 60–100 crystals in the same interval), with lower biocementation captured for the first 200 mm.

### Evaluation of permeability

The permeability of the microfluidic channels was measured during CS injection. By rearranging Darcy’s law for incompressible flow in terms of the intrinsic permeability k (m^2^), then:

6 $$k = \frac{Q\mu L}{{A\left( {p_{1} - p_{2} } \right)}}$$

where $Q$ is the imposed flow rate (m^3^/s), $\mu$ is the water dynamic viscosity (N∙s/m), L (m) is the length of the microfluidic channel, A (m^2^) is the cross-sectional area (HxW), $p_{1}$ (N/m^2^) is the measured pressure at the inlet and $p_{2}$ (N/m^2^) is the measured pressure at the outlet.

**Figure 12:**
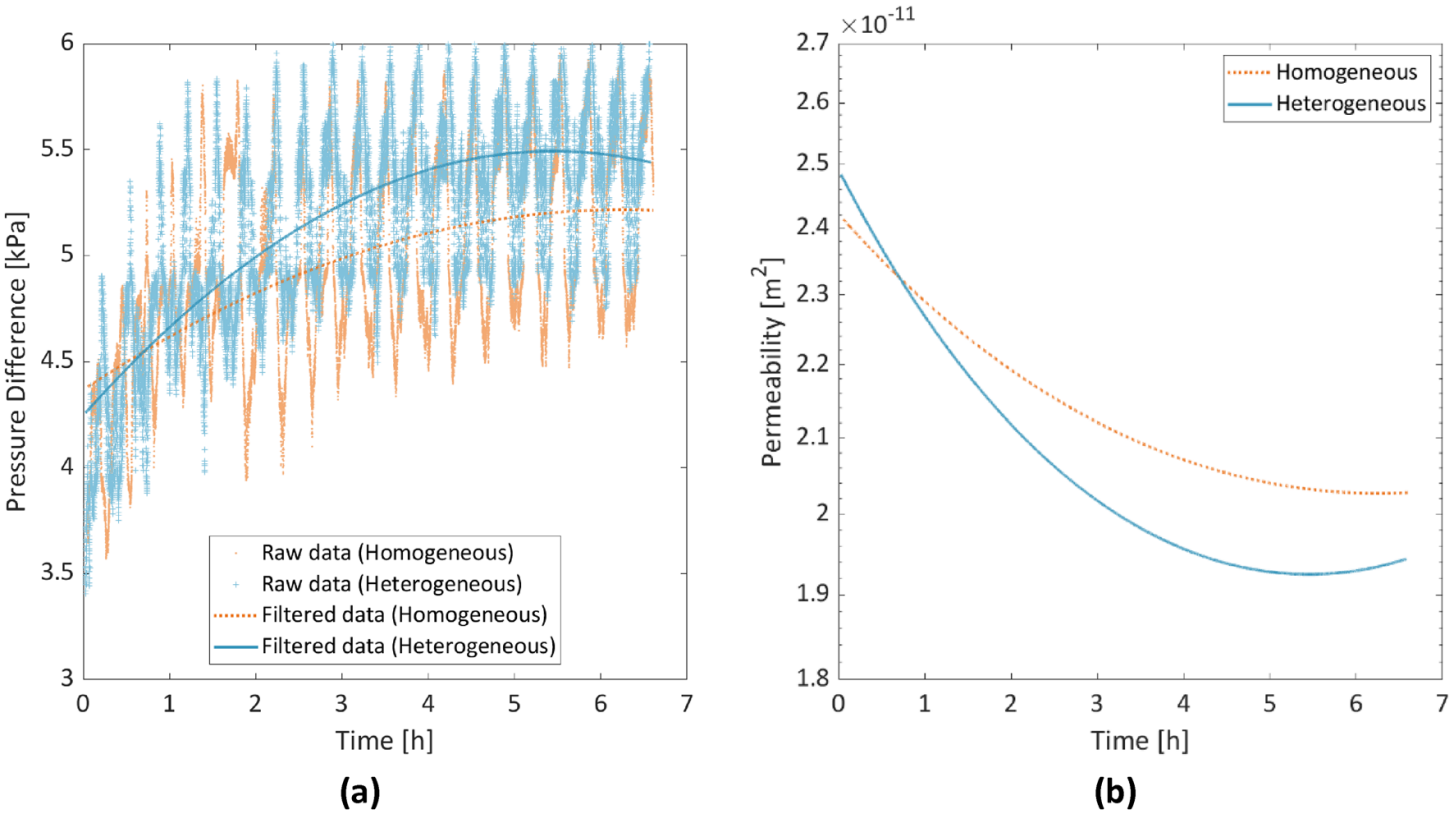
(**a**) Raw and filtered data (with the Savitzky-Golay filter^45^) of pressure difference evolution. (**b**) Calculated intrinsic permeability evolution during CS injection.

The CS injection during which crystals were formed and grew lasted as aforementioned for 6 h and 40 min. Figure 12 shows the measured pressure difference between the inlet and the outlet during the CS injection for the 3rd replicate of the experiment in the homogeneous and the heterogeneous porous medium. The raw data were smoothed with the use of the Savitzky-Golay filter^45^ in Matlab, which is a moving window technique that is based on local least squares polynomial fitting across the time domain. The pressure sensors were able to capture the increase in pressure difference due to the reduction in porosity by crystal nucleation and growth, which reached an almost steady state 4–5 h later. This is in agreement with the observed trend regarding the crystal growth both qualitatively (Figs. 7, 8) and quantitatively (Fig. 9) that most of the crystal growth occurs in the first 4–5 h of the CS injection. Figure 12 shows the derived evolution of the intrinsic permeability with the use of Eq. (6).

After the end of the CS injection, a 16% reduction in the permeability of the homogeneous porous medium was observed, while for the heterogeneous porous medium, the reduction was 22%. The difference in permeability reduction between the two chips is only 6%, which implies that the microscopic difference in the size and number of crystals, does not affect predominantly the macroscopic permeability. The low permeability reduction shows that there are enough alterative flow paths that are not clogged. The overall changes in permeability remain within the expected range of 1 order of magnitude^19^. CaCO_3_ precipitation in narrow pore throats might increase the mechanical strength and stiffness^25,46^, in contrast to precipitation in open pore throats that might efficiently reduce the permeability^19,25^.

## Conclusions

We monitored and visualized MICP in real-time within homogeneous and heterogeneous chips. A novel experimental setup for the evaluation of the permeability changes during the MICP treatment and efficiency calculations was presented in this work. The number of bacteria and the mass of precipitates at different positions along the trajectories were quantified to express the calcite distribution and reaction efficiency and ultimately determine the effect of the pore networks on the precipitation patterns. The work introduced a MATLAB-based image processing workflow to facilitate the handling of a large amount of data produced by comprehensive image acquisition. The major conclusions from this work include the following:

- With a continuous injection of 6 pore volumes of CS, CaCO_3_ crystals were formed 1 h after the beginning of the injection of CS and completed 12 h later in both microfluidic channels.
- The crystal growth in terms of the produced mass of CaCO_3_ at different positions along the chip is overall higher in the heterogeneous porous medium considering that identical treatment conditions were applied.
- By using opposite ends for the introduction of BS and CS, in the heterogeneous porous medium, biocementation increased to 34% at the middle of the chip (420 mm) and subsequently evolved toward the CS flow path with values above 20%, while in the homogeneous porous medium, a peak of 27% was reached 420 mm after the CS injection point, and the reaction efficiency remained less than 12% lower in the rest of the microfluidic medium. This is attributed to the observed trends of a higher number of crystals that grow in diameter despite quasi-identical initial bacterial distribution for both media. This implies that the dynamic evolution of reactive flow path is a key source towards understanding the final evolution of MICP-induced CaCO_3_ deposit
- The above findings are captured after comparing triplicates where the initial treatment conditions (OD, flow parameters, injection setup) were identical prior to injection into the homogeneous and heterogeneous chips. In the homogeneous porous medium, the average velocity is uniform, leading to a uniform bacterial distribution. In contrast, in the heterogeneous porous medium, which consists of zones of higher and lower velocity, more bacteria accumulate in the second half of the chip, which also possibly offers more available nucleation sites for the crystals. This suggests that the intrinsic properties of the porous materials and the architecture of their porous networks influence the fate of precipitation and the tortuous reactive paths. Our study sheds new light on the evolution of MICP and its adaptation to the available flow network by harnessing technological advances in microscopy, large data analysis and microfluidic devices, fields that are expected to grow within the traditional area of geomechanics.
- The proposed experimental setup and image analysis framework can be extended to the real-time monitoring of MICP via multiple treatment patterns using different recipes with the use of microvolumes.

## Supplementary Information

Supplementary Information.

## Data Availability

Datasets and code used for this study will become available upon publication under DOI: 10.5281/zenodo.7319582.
