# Supplementary material for: Microfluidic study in a meter-long reactive path reveals how the medium’s structural heterogeneity shapes MICP-induced biocementation
Source: Sci Rep. 2022 Nov 15;12:19553. doi: 10.1038/s41598-022-24124-6 (PMC9666553; doi:10.1038/s41598-022-24124-6)
Supplement: Supplementary file 1 — Supplementary Information. [file 41598_2022_24124_MOESM1_ESM.docx]

# Supplementary information

**Table S1.** Calculation of Reynolds number in the homogeneous and heterogeneous chips

|  | **Homogeneous chip** | **Heterogeneous chip** |
| --- | --- | --- |
| **ρ [kg·m^-3^]** | 997 | 997 |
| $\bar{\boldsymbol{u}}$ **[m s^-1^]** | 1.20E-04 | 1.20E-04 |
| $\bar{\boldsymbol{\lambda}}$ **[m]** | 0.00005 | 0.00007 |
| **μ [kg·m^-1^·s^-1^]** | 8.90E-04 | 8.90E-04 |
| **Re [-]** | 6.72E-03 | 9.41E-03 |

| 1. Red Channel of Phase Contrast Image   Index=0.01176 |
| --- |
| 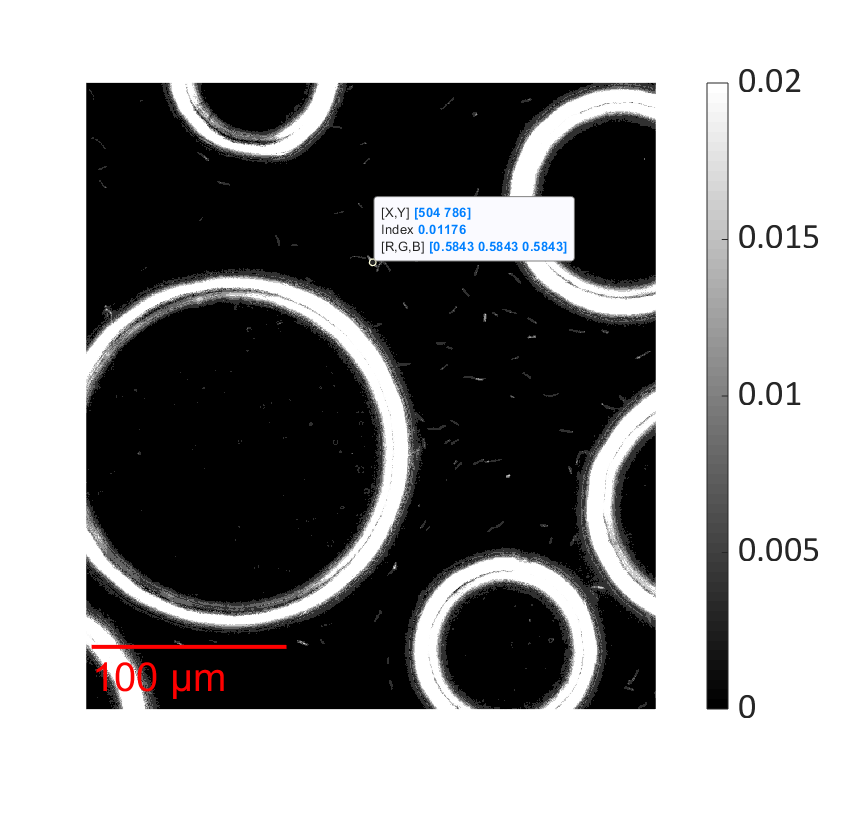 |
| 1. Green Channel of Phase Contrast Image   Index=0.03137 |
| 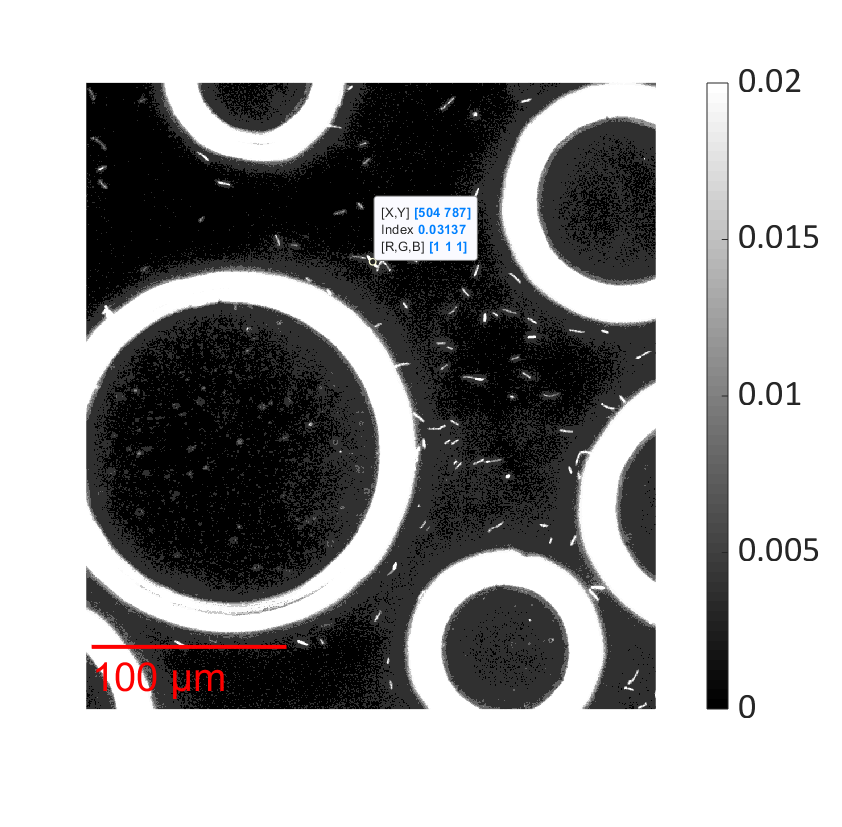 |
| 1. Blue Channel of Phase Contrast Image   Index=0.003922 |
| 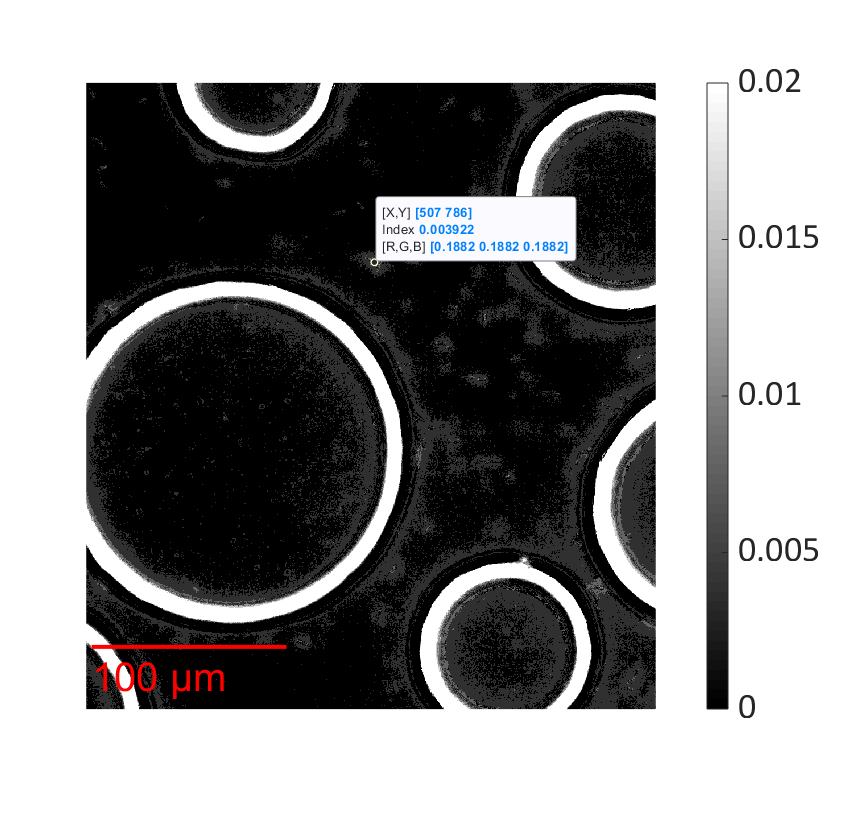 |

**Figure S1.** **(a)** Red, **(b)** green, **(c)** blue channel of the Cropped Phase Contrast Image of dimensions 0.292 mm x 0.321 mm (W x L), where the intensity of the same cell is compared. The pixel values were normalized by dividing with 255 to acquire pixel values between 0 and 1.

| **(a)** Red Channel of Brightfield Image |
| --- |
| 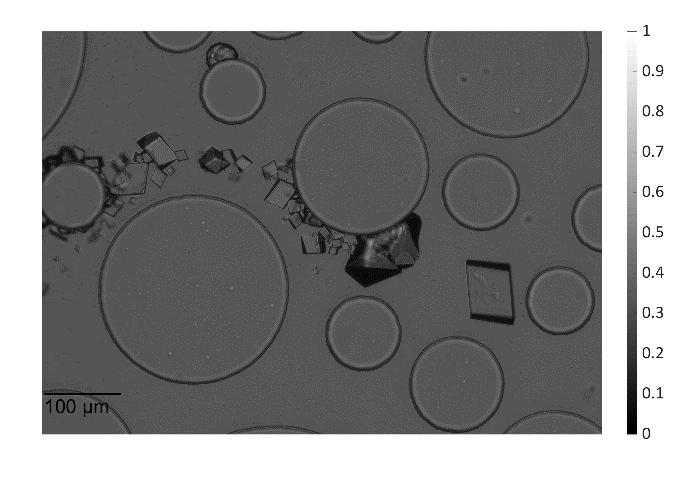 |
| **(b)** Green Channel of Brightfield Image |
| 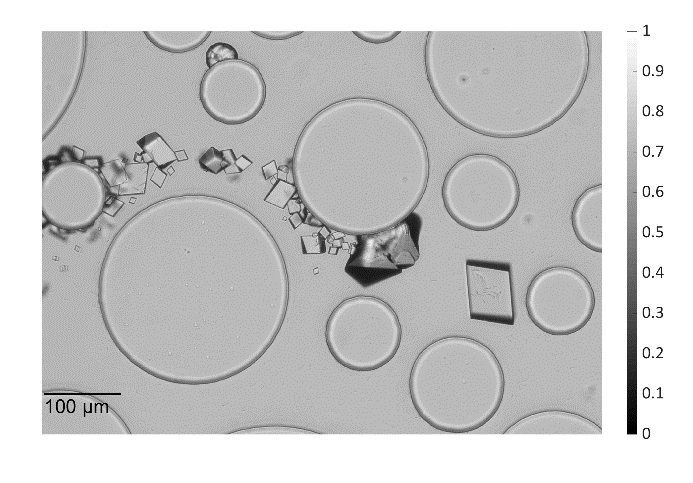 |
| **(c)** Blue Channel of Brightfield Image |
| 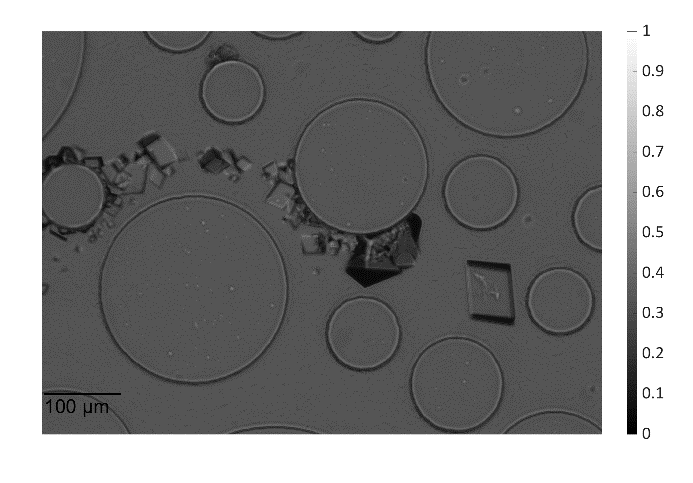 |

**Figure S2.** **(a)** Red, **(b)** green, **(c)** blue channel of Cropped Brightfield Image of dimensions 0.73 mm x 0.53 mm (W x L). The pixel values were normalized by dividing with 255 to acquire pixel values between 0 and 1.

**Bacterial attachment and aggregation**


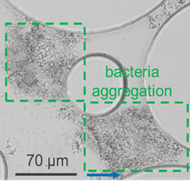


**Figure S3**. Example of bacterial aggregation at the side of injection of CS hindering the counting of single bacteria at the closest position to the inlet.


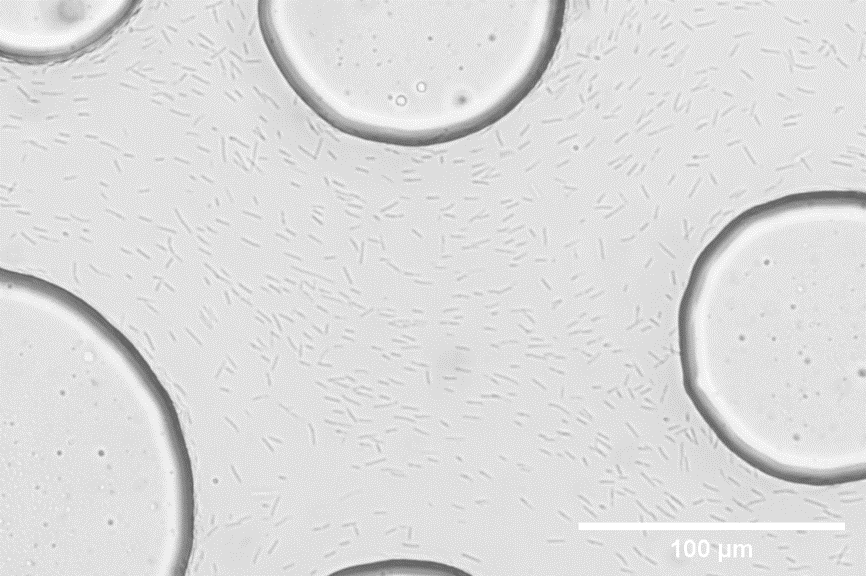


**Figure S4.** Single cells and cells that grew by cell division in the heterogeneous porous medium at the beginning of the CS injection (t=0) ∼ 492 mm from the inlet. This is a cropped area of the green channel of the original image captured with brightfield microscopy.

**Separation of solid grains: Selection of the value of “Sensitivity” parameter for “imbinarize” function**

The threshold was determined by the “Sensitivity” parameter, which can take values from 0 to 1 (see Supplementary material). By using a higher sensitivity, more pixels are defined as outlines of the pillars. For the most sampled positions in the 6 experiments, the “Sensitivity” was set to the default value of 0.50, while for a few positions, it was set between 0.52-0.58 when the outlines of all pillars were not detected with the use of the default value.

**Separation of precipitated CaCO_3_: Filtering of pixels that correspond to outlines of pillars**

Due to changes in illumination during the experiment, the outline of some pillars remained, and additional steps were required to remove them so that they would not be accounted for in the volume of the precipitated CaCO_3_. These remaining pixels were characterized by a high proportion of the length of the major axis to the length of the minor axis and a lower axis length compared to the crystals. Thereby, they were identified with the use of the "regionprops" MATLAB command. "Salt and pepper" noise was added with the MATLAB function "imnoise" to 5% of the pixels of the image, which means that random black and white pixels were added to the image matrix, and median filtering (“medfilt2” function) was used to denoise the image. For the median filtering (“medfilt2”), a square object of a maximum size of 10 pixels by 10 pixels was used, which resulted in the removal of some tiny crystals of this size

**Example of processed large images**

| 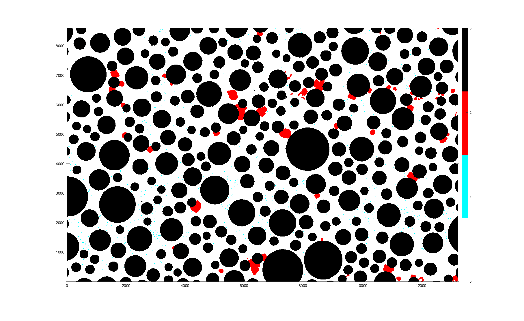 **(a)** | 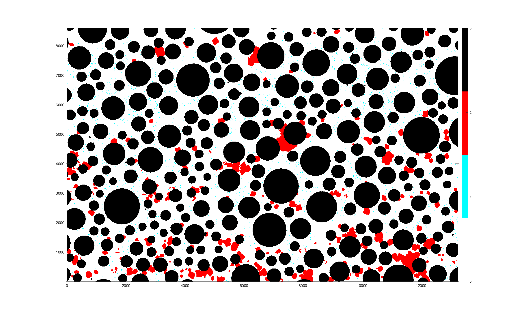 **(b)** | 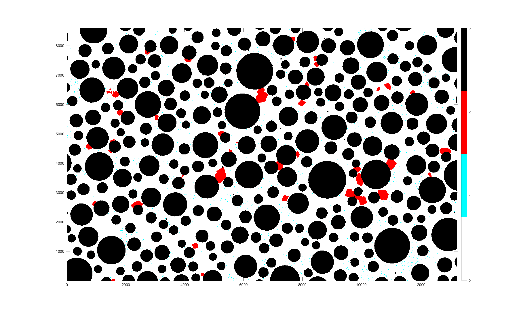  **(c)** |
| --- | --- | --- |

**Figure S5.** Processed large images of 3.9 mm x 2.6 mm captured at sample positions **(a)** 340 mm, **(b)** 425 mm and **(c)** 490 mm downstream from the inlet 12 hours after the beginning of CS injection. Red: precipitated CaCO_3_, Black: solid grains. Cyan: bacteria
